# Mitochondrial DNA mutation screening of male patients with obstructive sleep apnea-hypopnea syndrome

**DOI:** 10.3892/etm.2014.1748

**Published:** 2014-06-04

**Authors:** XIAO-YING HUANG, HONG LI, XIAO-MEI XU, LIANG-XING WANG

**Affiliations:** 1 Department of Respiratory Medicine, The First Affiliated Hospital of Wenzhou Medical University, Wenzhou, Zhejiang 325003, P.R. China; 2 Department of Respiratory Disease, Hospital of Huabei Petroleum Administration Bureau, Renqiu, Hebei 062552, P.R. China

**Keywords:** mitochondrial DNA, obstructive sleep apnea-hypopnea syndrome, genovariation, polymorphism

## Abstract

The aim of the present study was to analyze the differences between the genes of the mitochondrial DNA (mtDNA) displacement loop (D-loop) region and the Cambridge Reference sequence, in order to screen the mutation sites and investigate the correlation between mutations, clinical parameters and complications associated with obstructive sleep apnea-hypopnea syndrome (OSAHS). mtDNA was obtained from male patients with OSAHS in the Zhejiang Province. In total, 60 male patients with OSAHS and 102 healthy adults were assessed to determine the levels of fasting blood glucose, total cholesterol, triglyceride (TG) and high-density and low-density lipoproteins (LDL). Furthermore, peripheral mtDNA was extracted and bidirectional sequencing was conducted to enable mutation screening. In the mtDNA D-loop region, 178 mutation sites were identified, of which 115 sites were present in the two groups. The number of non-common sites in the OSAHS group was significantly higher compared with the control group (P<0.05). No statistically significant difference was observed in the mutations among the mild, moderate and severe OSAHS groups (P>0.05). A total of 21 cases in the severe OSAHS group exhibited mutation rates of >10%. In the control group, there were 24 cases where the np73A-G and np263A-G mutations were predominant. The np303–np315 region was identified to be the highly variable region and various mutation forms were observed. Statistically significant differences were observed in the neck perimeter, TG and LDL levels among the OSAHS-no-mutation subgroups (P<0.05) and LDL was shown to be associated with an mtDNA mutation in the OSAHS group. Numerous polymorphic mutation sites were identified in the mtDNA D-loop region of the OSAHS group. Therefore, mtDNA mutation sites may be closely associated with the clinical manifestations and complications of OSAHS.

## Introduction

Sleep apnea-hypopnea syndrome (SAHS) can be divided into three types: Obstructive, central and mixed. In clinical practice, obstructive SAHS (OSAHS) is one of the most common forms, accounting for ~80% of SAHS cases. Previous studies have identified that the incidence rate of OSAHS in adults is 1–5% (1) or 41% in a subpopulation of patients with a body mass index (BMI) of >28 kg/m^2^ (2). The incidence rate of domestic OSAHS is ~3%. Furthermore, SAHS is an independent risk factor of multiple systemic diseases (3,4) and seriously affects patient health and quality of life. The pathogenesis of SAHS remains unclear, however, previous studies have demonstrated familial aggregation of OSAHS and genetic predisposition (5). Patients with OSAHS suffer from recurrent nocturnal intermittent hypoxia, which affects oxidative phosphorylation in the mitochondrial respiratory chain. Obesity, hyperlipidemia, hyperglycemia and other pathogenesis factors are closely associated with OSAHS, and may be associated with mitochondrial mutations (6,7).

In addition to the nucleus in human cells, the mitochondrion is an organelle that contains genetic material. Each mitochondrion contains 2^10^ copies of mitochondrial DNA (mtDNA) and is able to perform replication, transcription and translation without relying on nuclear DNA (nDNA). The half-life of mtDNA is 5–10 times faster than nDNA and the mutation rate of mtDNA is 10–20 times higher compared with nDNA (8–11). Thus, mtDNA exhibits greater vulnerability and is damaged more frequently compared with nDNA; in addition, mtDNA possesses a greater risk of mutation than nDNA. The displacement loop (D-loop) is the control region of mtDNA replication and is frequently mutated. The majority of the regulatory sequences associated with mtDNA replication, transcription and translation are located within the D-loop (12). The aim of the present study was to investigate the correlation between mitochondrial mutations and OSAHS, as well as the associated complications, and to investigate whether the genetic predisposition of OSAHS is associated with mitochondrial mutations.

## Patients and methods

### Subjects

In total, 60 male patients with OSAHS and 102 healthy males were recruited from the Sleeping Center of the First Affiliated Hospital of Wenzhou Medical University (Wenzhou*, China) between June 2007 and December 2007.* The subjects were not related to one another and those presenting with the following conditions were excluded: Glucose metabolism disorder, lipid metabolism disorder, endocrine disorder, chronic obstructive pulmonary disease, heart failure, jaundice or central nervous system diseases. The age and gender of the two groups were matched. The study was conducted in accordance with the Declaration of Helsinki and with approval from the Ethics Committee of Wenzhou Medical University. Written informed consent was obtained from all the participants.

### Clinical data collection

Clinical symptoms, including height, weight, neck perimeters, chest and hip complications, were recorded in each subject.

### Biochemical parameter detection

Routine biochemical parameters, including levels of fasting blood glucose (FBG), triglyceride (TG), total cholesterol (TC), high-density lipoprotein (HDL) and low-density lipoprotein (LDL), were measured for each subject.

### Genomic DNA extraction

Peripheral blood samples were collected from the patients and healthy subjects using a K2-EDTA anticoagulant. The genomic DNA extraction kit (Universal Genomic DNA extraction kit version 3.0; Takara Biotechnology Co., Ltd., Dalian, China) was used to extract the peripheral blood genomic DNA, according to the manufacturer’s instructions.

### Polymerase chain reaction (PCR)

PCR reagents and primers, including *Taq* DNA polymerase and a DNA fragment purification kit, were purchased from Takara Biotechnology Co., Ltd.

The D-loop region of the mitochondrial gene was located at mtDNA np16028-577 and two pairs of primers, with overlapping product regions, were used to amplify the genes of the entire D-loop region. The sequences and amplified products of the two pairs of primers are shown in Table I. In addition, when designing the two pairs of primers, one universal M13 forward primer, TGTAAAACGACGGCCAGT and one reverse primer, CAGGAAACAGCTATGACC, were synthesized at the 5′ end. The PCR conditions were as follows: 35 cycles of predenaturation at 94°C for 5 min, denaturation at 94°C for 30 sec, annealing at 59°C for 45 sec and extension at 72°C for 1 min, followed by a final extension at 72°C for 5 min. Next, 3 μl PCR product was mixed with 1 μl 6× loading buffer and the mixture was added into a gel well. A 2,000 bp DNA marker (3 μl) was then added and electrophoresis was performed at a voltage of 5 V/cm for 30 min. The gel was photographed using an imager camera and the DNA fragment purification kit was used for purification, according to the manufacturer’s instructions.

### DNA sequence analysis

Purified PCR products were subjected to DNA sequence analysis (Shanghai GeneCore BioTechnologies Co., Ltd., Shanghai, China). The SeqMan function of the DNASTAR software package (DNASTAR, Inc., Madison, WI, USA) was used for sequence analysis and the MegAlign function (DNASTAR, Inc.) was used to compare the products with the reference sequence (human mtDNA revised Cambridge reference sequence) in order to identify the mutation locus. When the potential mutation sites were identified, Chromas software (Technelysium, Brisbane, Australia) was used to observe the specific peaks, and compare and analyze the sites with the reported mtDNA mutation sites in the internationally recognized human mitochondrial database, (www.mitomp.org).

### Statistical analysis

SPSS 13.0 statistical analysis software (SPSS, Inc., Chicago, IL, USA) was used for analysis and data are expressed as the mean ± standard deviation. Inter-group comparisons of normally distributed data were performed using the Student’s t-test, whereas logistic regression analysis with a bilateral statistical test level (P<0.05) was used for analyzing the independent variables, such as the clinical data.

## Results

### Genomic DNA extraction

**Figure 1:**
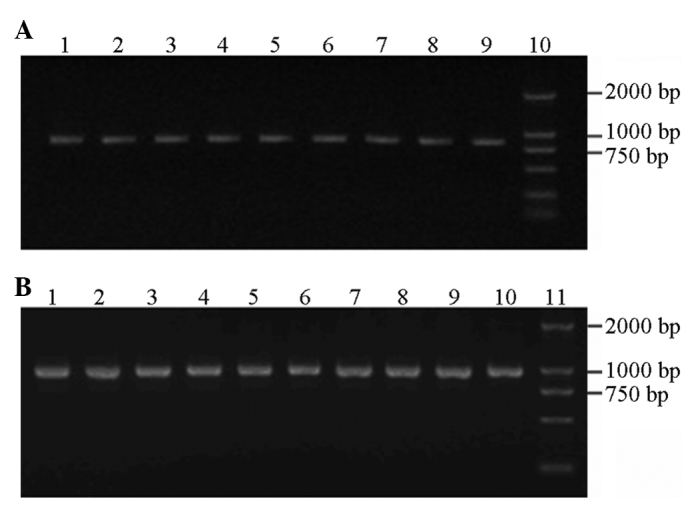
PCR products were detected by agarose gel electrophoresis. (A) Lanes 1–9, PCR products of the primers of 23rd site of mitochondrial DNA (length, 764 bp); lane 10, DL2000 DNA marker. (B) Lanes 1–10, PCR products of the primers of 24th site of mitochondrial DNA (length, 925 bp); lane 11, DL2000 DNA marker. PCR, polymerase chain reaction.

Following extraction and purification of the peripheral blood DNA, a clearly visible band was observed in the 1.0% agarose gel electrophoresis image (Fig. 1).

### PCR

Agarose gel (1.2%) electrophoresis was performed with a 2,000 bp DNA ladder marker that served as the molecular weight standard to identify the amplification products. The *in vitro* amplification of the target genes obtained two fragments with lengths of 764 and 925 bp (Fig. 1).

### Single nucleotide polymorphism

Mutation sites were identified in the mtDNA D-loop region of each sample (Table II). A total of 178 mutation sites were found, of which 75 mutation sites were identified in the 60 OSAHS subjects and 103 mutation sites were identified in the 102 healthy control subjects. A total of 115 mutation sites were present in the two groups and the 63 remaining mutation sites were not considered to be common. A total of 27 mutation sites were present only in the OSAHS group, whereas 36 mutation sites were present only in the healthy control group, which exhibited a low incidence rate of mutation. The distribution of the single-mutation sites in the OSAHS and healthy control groups did not demonstrate a statistically significant difference (P>0.05). However, for the non-common mutation sites, a significant difference was identified between the OSAHS group and the normal control group (P<0.05). No significant differences in the mutation sites were observed among the mild, moderate and severe OSAHS subgroups (P>0.05).

### High-mutation sites

**Figure 2:**
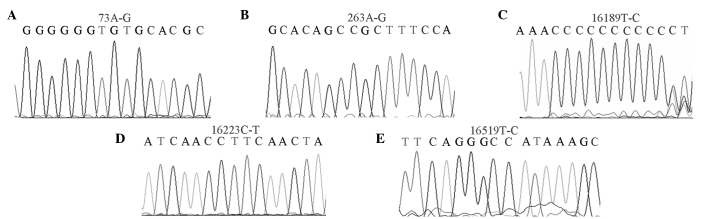
High-mutation sites in the D-loop region, including (A) 73A-G, (B) 263A-G, (C) 16189T-C, (D) 16223C-T and (E) 16519T-C mutations. D-loop, displacement loop.

In total, 21 high-mutation sites (mutation rate >10%) were found in the OSAHS group, whereas 24 sites were identified in the healthy control group (Fig. 2). The np73A-G mutation rates were 93.7 and 84.2%; np263A-G mutation rates were 90.4 and 79.3%; np16223C-T mutation rates were 50.1 and 47.0%; np16189T-C mutation rates were 46.7 and 29%; and np16519T-C mutation rates were 53.3 and 46% in the OSAHS and healthy control groups, respectively.

### Mutation sites of np303–np315

**Figure 3:**
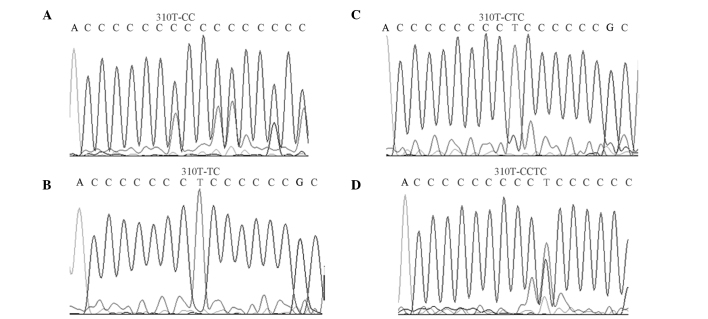
Mutations in the D310 region, including (A) T-CC, (B) T-CTC, (C) T-TC and (D) T-CCTC mutations.

The mutation site of np303–np315, known as the D310 region, has a high mutation rate. The mutation rate in the D310 region of the OSAHS group was significantly higher compared with the healthy group (P<0.05; Fig. 3).

No statistically significant difference was identified in the clinically relevant data (including Rohrer index) between the mutation and non-mutation groups in the D310 region of the OSAHS group (P>0.05; Table III).

### Mutation rates of np16189T-C

Mutation rates of np16189T-C in the OSAHS and control groups were 46.7 and 29%, respectively. Statistical significance was observed between the np16189T-C mutation and the neck perimeter, TG and LDL levels in the OSAHS group (P<0.05; Table IV).

### Logistic regression analysis

Logistic regression analysis of the clinical characteristics of the mtDNA mutations in the OSAHS group indicated that LDL-cholesterol was associated with a genetic mutation (Table V).

## Discussion

OSAHS has a number of causes and influencing factors, including obesity, hyperlipidemia and hyperglycemia, which are also associated with mitochondrial mutations (13,14).

The D-loop region is the starting point of mtDNA replication and the site of regulatory functions in mtDNA transcription and replication for the major non-coding regions of human mtDNA. Therefore, mutations in the D-loop region lead to disorders in mitochondrial function. Numerous diseases have been reported to be associated with mutations in the D-loop region of mtRNA, and this region is considered to be the common site of DNA mutations in various gene-associated diseases (15). Therefore, two pairs of primers that covered the mtDNA D-loop region were selected for amplification and sequencing, and compared and analyzed with the standard Cambridge sequence to investigate the correlation between the polymorphisms in the D-loop region of the mitochondrial genome with the occurrence, clinical manifestations and complications associated with OSAHS.

Compared with the Cambridge standard sequence, mutation sites were identified in the mtDNA D-loop region in each sample from the OSAHS and healthy control groups. A total of 178 mutation sites were found, of which 75 mutation sites were observed in the 60 OSAHS cases and 103 mutation sites were found in the 102 healthy control subjects. In total, 115 mutation sites were present in the two groups; 27 mutation sites were present only in the OSAHS group and 36 mutation sites were present only in the healthy control group. Statistical analysis indicated that the non-common mutation sites found only in the OSAHS group were significantly greater than those in the control group, indicating that the mtDNA D-loop region is a high-mutation-incidence region. This observation is consistent with previous literature (16).

These results indicated that the human mtDNA standard sequences, when compared between a Zhejiang Han population and a Western Caucasian population, were not homologous. This difference may be due to genetic polymorphisms, which may be determined by the structural characteristics of a particular population. The D-loop region is the site where mtDNA connects with the inner mitochondrial membrane, thus, the D-loop region is increasingly susceptible to peroxide damage. During mtDNA replication, the D-loop region forms a three-chain structure that exhibits greater susceptibility to injury (17). The presence of numerous mutations in the mtDNA D-loop region indicates that mitochondrial oxidative stress results in mutations (18). Although the mtDNA D-loop region is the non-coding region of the mitochondrial genome, the region contains essential elements for transcription and replication. Thus, mutations in the D-loop region may affect the transcription and replication of mtDNA. Furthermore, such mutations can occur in the coding regions through multiple-site mutations in the D-loop region, which would control and change the transcription and translation of the coding genes. Thus, the occurrence and development of diseases may be affected.

The D310 region consists of 12–18 cytosine residues and contains the highest number of mitochondrial microsatellite polymorphisms, thus, is the major microsatellite-unstable region of mtDNA (19). In addition, the D310 region is responsible for the formation of RNA-DNA complexes that initiate mtDNA replication (20). In mitochondria-associated diseases, the repeated deletion and insertion of a single type of nucleotide (polyC) between np303 and np310 in the D-loop region has become a major research focus. In the present study, the mutations in the D310 region in the OSAHS group were significantly higher compared with the healthy control group, indicating that the mutations in this region may increase OSAHS susceptibility. In addition, a greater quantity of mutation sites with higher frequencies were observed in the OSAHS group. OSAHS is a chronic disease with multiple causative genes, therefore, further investigation is required to determine whether the D-loop region, as the control region, contains numerous mutation sites that affect the mutation of the entire mitochondrial genome.

In the OSAHS group, the relevant clinical parameters included age, BMI, neck perimeter, FBG, TC, TG, HDL and LDL levels. The clinical data, with or without mitochondrial mutations, were compared in the OSAHS group and the results revealed statistically significant differences in the neck perimeter and TG and LDL levels between the two groups (P<0.05). In addition, logistic regression analysis was performed on partial clinical characteristic variables of the patients with mitochondrial mutations in the OSAHS group, and the results indicated that the LDL level was associated with gene mutation.

Analysis of the mutation sites revealed 21 high-mutation sites (mutation rate >10%) in the OSAHS group and 24 sites in the healthy control group; the mutation rates of np16519T-C in the OSAHS and control groups were 53.3 and 46%, respectively. Poulton *et al* (21) hypothesized that the mitochondrial 16189 mutation was the candidate gene of the thrifty genome, thus, a patient with a 16189 mutation experiences an increased risk of obesity, diabetes and other diseases with increasing age. This mutation site was considered to promote adult insulin resistance and contribute significantly to the development of non-insulin-dependent diabetes (22). Furthermore, Lin *et al* (23) indicated that the np16189T-C mutation reduced antioxidant functions under stress, thus, this mutation increased the susceptibility to diabetes and exacerbated the diabetic condition. OSAHS has been demonstrated to be associated with risk factors of obesity, hypertension, insulin resistance and high cholesterol levels. In particular, obesity is the predominant risk factor of OSAHS, and OSAHS and obesity susceptibility may have a common function through specific common genes (24). The present study revealed that the 16189T-C mutation rate of OSAHS patients, excluding the risk of endocrine disorders, was significantly higher compared with the healthy group, whereas no statistically significant difference was identified among the mild, moderate and severe subgroups of OSAHS. Therefore, the insulin resistance of OSAHS patients is likely to be greater than the non-OSAHS group, however, the occurrence rate of insulin resistance is not associated with the severity of OSAHS. The incidence and associated complications of OSAHS patients correlate with the 16189T-C mutation in mtDNA.

In conclusion, the present study demonstrated that specific sites in the D-loop region are associated with certain clinical symptoms and complications in patients with OSAHS. The observations emphasize the importance of early detection, prevention and treatment, and complication reduction in patients with OSAHS.

## Figures and Tables

**Table I:** Primers used to amplify the genes of the mitochondrial D-loop region.

Primer	Product sequence (5′-3′)		Location of 3′ (F/R)	Product length (bp)

	F	R		
Mit-23	TCATTGGACAAGTAGCATCC	GAGTGGTTAATAGGGTGATAG	15811/5	764
Mit-24	CACCATTCTCCGTGAAATCA^a^	AGGCTAAGCGTTTTGAGCTG	16420/775	925

a Overlapping fragments at 155.

F, forward; R, reverse; Mit-23, primers of 23rd site of mitochondrial DNA; Mit-24, primers of 24th site of mitochondrial DNA.

**Table II:** Mutation sites in the mtDNA D-loop region of the OSAHS patients and control group.

Mutation sites	Nucleotide change	OSAHS group (n)	Control group (n)
73	A-G	57	86
103	G-A	2	1
146	T-A	4	0
150	C-T	8	16
198	C-T	2	1
204	T-C	2	5
214	A-G	2	1
249 delA	7	21	
263	A-G	55	89
298	C-T	1	1
310	T-CTC	16	22
310	T-CC	8	11
16140	T-C	7	4
16164	A-G	3	4
16316	A-G	3	1
16325	T-C	3	1
16327	C-T	2	2
16357	T-C	2	1
16362	T-C	11	34
16182	A-C	8	12
16183	A-C	12	23
16184	C-T	7	7
16185	C-T	3	2
16189	T-C	16	30
16192	C-T	2	2
16217	T-C	2	4
16223	C-T	31	46
16243	T-C	4	1
16249	T-C	2	1
16260	C-T	2	7
16266	C-T	1	3
16172	T-C	2	19
16319	G-A	8	12
16390	G-A	2	2
16463	A-G	2	1
16519	T-C	32	47
16535	G-A	1	0

OSAHS, obstructive sleep apnea-hypopnea syndrome; mtDNA, mitochondrial DNA; D-loop, displacement loop.

**Table III:** Analysis of D310 regional mutations with clinical and biochemical indicators in the OSAHS group.

Indicator	Mutation group (n=28)	No mutation group (n=32)	P-value
Age	39.77±11.35	40.47±11.27	>0.05
BMI	25.86±2.77	26.52±2.28	>0.05
Rohrer index	162.90±33.40	150.50±17.50	>0.05
Neck collar	41.30±2.43	34.50±2.20	>0.05
FBG	4.95±0.48	4.86±0.52	>0.05
TC	5.64±0.96	5.46±0.96	>0.05
TG	3.81±2.44	3.39±1.35	>0.05
HDL-C	1.13±0.24	1.16±0.22	>0.05
LDL-C	2.78±0.62	2.66±0.54	>0.05

OSAHS, obstructive sleep apnea-hypopnea syndrome; BMI, body mass index; FBG, fasting blood glucose; TC, total cholesterol; TG, triglyceride; HDL, high-density lipoprotein-cholesterol; LDL, low-density lipoprotein-cholesterol.

**Table IV:** Analysis of the np16189T-C mutation with clinical and biochemical indicators in the OSAHS group.

Indicator	Mutation group (n=28)	No mutation group (n=32)	P-value
Age	38.40±11.65	41.44±10.83	>0.05
BMI	25.82±2.85	26.33±2.40	>0.05
Rohrer index	158.90±37.40	144.50±18.50	>0.05
Neck collar	40.30±2.60	38.50±2.40	<0.05
FBG	4.90±0.50	4.95±0.50	>0.05
TC	5.67±0.97	5.48±0.95	>0.05
TG	3.74±2.73	3.73±1.67	<0.05
HDL-C	1.09±0.24	1.18±0.23	>0.05
LDL-C	2.29±0.37	3.18±0.52	<0.05

OSAHS, obstructive sleep apnea-hypopnea syndrome; BMI, body mass index; FBG, fasting blood glucose; TC, total cholesterol; TG, triglyceride; HDL, high-density lipoprotein-cholesterol; LDL, low-density lipoprotein-cholesterol.

**Table V:** Logistic regression analysis between clinical features and mutations in the OSAHS group.

Characteristic	Score	P-value
BMI	0.307	0.579
Smoking	0.576	0.448
Drinking	1.603	0.206
HBP	0.066	0.798
TC	1.037	0.309
TG	1.152	0.697
HDL-C	0.152	0.697
LDL-C	4.443	0.035

OSAHS, obstructive sleep apnea-hypopnea syndrome; BMI, body mass index; HBP, high blood pressure; TC, total cholesterol; TG, triglyceride; HDL, high-density lipoprotein-cholesterol; LDL, low-density lipoprotein-cholesterol.
